# Novel rapid-immunohistochemistry using an alternating current electric field for intraoperative diagnosis of sentinel lymph nodes in breast cancer

**DOI:** 10.1038/s41598-017-02883-x

**Published:** 2017-06-05

**Authors:** Kaori Terata, Hajime Saito, Hiroshi Nanjo, Yuko Hiroshima, Satoru Ito, Kasumi Narita, Yoichi Akagami, Ryuta Nakamura, Hayato Konno, Aki Ito, Satoru Motoyama, Yoshihiro Minamiya

**Affiliations:** 1 Department of Thoracic Surgery, Akita University Graduate School of Medicine, Akita, Japan; 2 Department of Pathology, Akita University Hospital, Akita, Japan; 3 Akita Prefectural Research and Development Center, Akita, Japan

## Abstract

Axillary lymph node status and pathological diagnosis of sentinel lymph nodes (SLNs) is a prognostic factor that influences management of postoperative therapy. Recent reports indicate that one-step nucleic acid amplification and hematoxylin and eosin (HE)-stained frozen sections are effective for intraoperative diagnosis of SLNs. In the present study, we report a rapid-immunohistochemical staining (R-IHC) method that enables intraoperative detection of SLN metastases within 16 min using an anti-cytokeratin antibody. This is the first report on SLN diagnosis using R-IHC in patients with breast cancer. We prospectively examined 160 dissected SLNs from 108 breast cancer patients who underwent surgery at our institute. The dissected SLNs were sectioned and conventionally stained with HE or immunohistochemically labeled with anti-cytokeratin antibody using R-IHC procedures. Intraoperative R-IHC analyses were completed within 16 min, after which diagnoses were made by two pathologists. The total time required for intraoperative diagnosis was about 20 min. In this study series, R-IHC detected four metastatic SLNs that were undetected using conventional HE staining (4/20, 20.0%). Compared with subsequent permanent diagnosis, R-IHC offered 95.2% sensitivity and 100% specificity. These findings indicate R-IHC is a clinically applicable technique that enables precise and quick intraoperative detection of micro- and macrometastasis in breast cancer.

## Introduction

Axillary lymph node status is the most important prognostic factors for patients with early breast cancer, and determining lymph node status is crucial when deciding whether to administer adjuvant systemic therapy^1, 2^. For that purpose, one-step nucleic acid amplification (OSNA) and hematoxylin and eosin (HE) staining of frozen sections are effective methods for making intraoperative diagnoses. OSNA, for example, has a low false positive rate and high specificity^3, 4^. On the other hand, patients assessed to be tumor-free using routine HE staining are often found to be sentinel lymph node (SLN)-positive using immunohistochemical staining (IHC) with an anti-cytokeratin antibody^1, 5^. Thus, use of IHC for diagnosis of SLN metastasis in patients with breast cancer enables detection of greater numbers of metastases^6^. But although IHC enables detection of more metastases, especially small-volume metastases and micrometastases, it has no impact on patient outcome, systemic treatment or radiotherapy^7, 8^. It is therefore unclear how IHC would contribute to axillary lymph node diagnosis, and so it is not generally required for pathological diagnoses. In addition, because the standard IHC protocol requires 2–4 hours to complete, its clinical application for intraoperative diagnosis is impractical. To solve this problem, we have developed a novel device that enables us to complete rapid IHC (R-IHC) analyses in about 20 min using an alternating current (AC) electric field. We previously demonstrated the utility of R-IHC for detecting lymph node metastasis in non-small cell lung cancer^9^ as well as in brain tumors, where Ki-67/MIB-1 and CD20 immunostaining of frozen sections is useful for intraoperative diagnosis of central nervous system tumors^10^. In this series, we applied R-IHC for intraoperative SLN biopsy in patients with breast cancer and investigated its accuracy, cost effectiveness and clinical applicability.

## Results

Using the R-IHC procedure outlined in Table 1, immunohistochemical analyses were completed within about 16 min, and cytokeratin-positive nodes were examined by two pathologists within about 20 min. It is noteworthy that the incubation times for the primary and secondary antibodies were only about 5 min each. The AC electric field effectively quickened the antigen-antibody reaction^9^.

**Table 1:** R-IHC procedure.

Procedures	Times (minutes)
Acetone fixation	0.5
Blocking endogenous peroxidase activity	1
Washing with phosphate-buffered saline	0.5
Primary antibody	5
Washing with phosphate-buffered saline	1
Simple stain MAX-PO (MULTI)®	5
Washing with phosphate-buffered saline	1
3,3′ diaminobenzidine	1
Washing with water	0.5
Hematoxylin nuclear counter staining	0.5
Approximate time required	16

The clinical characteristics of the study participants are summarized in Table 2. Seventy-one patients (65.7%) received breast-conserving surgery, while 37 (34.3%) underwent mastectomy. The histological types included 20 (18.5%) intraductal carcinomas with no metastases in SLNs. For the remaining nodes, the pathological classification was as follows: 90 (83.3%) were N0, 1 (0.9%) was N(i+), 4 (3.7%) were N1mi, 10 (9.3%) were N1a, and 3 (2.8%) were N2a. Thirty-two patients (29.6%) received chemotherapy (Farmorubicin, cyclophosphamide and/or fluorouracil; Taxotere or Taxol; Capecitabine; Trastuzumab), 79 (73.1%) received hormone treatment, and 60 (55.6%) received radiotherapy. Nine patients (8.3%) received neoadjuvant chemotherapy and they were initially lymph node-negative (cN0). HER2 status was determined immunohistochemically (Ventana I-VIEW PATHWAY; Roche Diagnostics) or by means of fluorescence *in situ* hybridization (FISH) using PathVysion Her2 DNA Probe kits (SRL Inc.). When a tumor showed +3 immunostaining, it was considered HER2-positive; when it showed 0 or +1 immunostaining, it was considered HER2-negative; when it showed +2 immunostaining, we applied FISH and if it contained more than two genes per cell, it was considered HER2-positive. Using these criteria, 19 (21.6%) patients were deemed HER2-positive.

**Table 2:** Clinical details of these breast cancer patients.

Characteristic		(n = 108)
Age		60 ± 14
Type of surgery	Breast-conserving surgery	71 (65.7%)
	Mastectomy	37 (34.3%)
Histological type	Invasive carcinoma NST	74 (68.5%)
	Lobular	4 (3.7%)
	Mucinous	8 (7.4%)
	Intraductal	20 (18.5%)
	Others	2 (1.9%)
Estrogen receptor status	Negative	18 (16.7%)
	Positive	90 (83.3%)
Progesterone receptor status	Negative	25 (23.1%)
	Positive	83 (76.9%)
Her2 status	Negative	69 (78.4%)
	Positive	19 (21.6%)
Pathological T classification	T0	3 (2.8%)
	Tis	20 (18.5%)
	T1	68 (62.9%)
	T2	15 (13.9%)
	T4	2 (1.9%)
Pathological N classification	N0	90 (83.3%)
	N0(i+)	1 (0.9%)
	N1mi	4 (3.7%)
	N1a	10 (9.3%)
	N2a	3 (2.8%)
Lymphatic invasion	No	49 (45.4%)
	Yes	59 (54.6%)
Vessel invasion	No	93 (86.1%)
	Yes	15 (13.9%)
Pathological staging	0	23 (21.3%)
	I	59 (54.6%)
	II	21 (19.5%)
	III	5 (4.6%)
Chemotherapy	No	76 (70.4%)
	Yes	32 (29.6%)
Hormone treatment	No	29 (26.9%)
	Yes	79 (73.1%)
Radiotherapy	No	48 (44.4%)
	Yes	60 (55.6%)
Neoadjuvant Chemotherapy	No	99 (91.7%)
	Yes	9 (8.3%)

Table 3 summarizes the metastasis diagnoses. We stained 160 axillary lymph nodes dissected as part of the surgical treatment for breast cancer. The R-IHC method detected four metastatic SLNs that were undetected using conventional HE staining (4/20:20.0%). In other words, all nodes deemed positive using the permanent (standard) IHC procedure were also positive using the R-IHC procedure. One lymph node (0.6%) containing ITCs was detected using permanent IHC in a permanent section, but not with R-IHC. However, this discovery did not change the histological node classification or the histological stage. Assuming the results obtained using R-IHC are correct, its sensitivity, specificity and accuracy were 95.2%, 100% and 99.4%, respectively, as compared with permanent IHC, while those values for intraoperative HE were 76.2%, 100%, 96.9%, respectively.

**Table 3:** Comparison of intraoperative and permanent pathological diagnoses of sentinel lymph nodes.

	Intraoperative		Permanent	
	HE Stain	R-IHC CK Stain	HE Stain	CK Stain
Macrometastasis	14 (8.8)	15 (9.4)	15 (9.4)	15 (9.4)
Micrometastasis	2 (1.3)	5 (3.1)	4 (2.5)	5 (3.1)
ITC	0	0	0	1 (0.6)
No Metastasis	144 (90.0)	140 (87.5)	141 (88.1)	139 (86.9)

**Figure 1:**
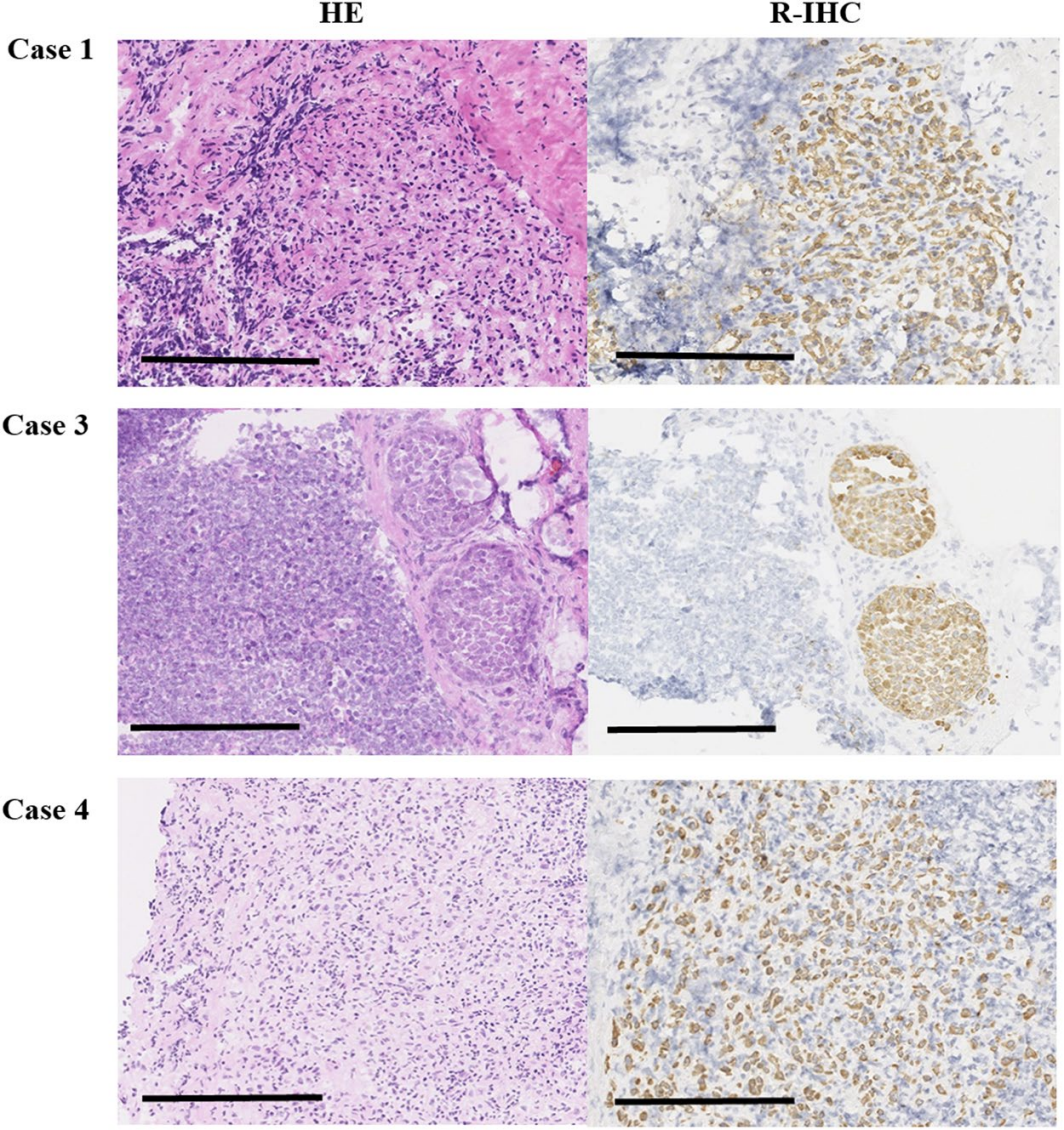
Images showing cytokeratin immunoreactivity within metastatic SLNs from the indicated cases. The tissue sections were stained with HE or with anti-cytokeratin antibody using R-IHC (bar = 200 µm).

Table 4 shows details of the cases in which the pathological diagnosis differed between the intraoperative HE staining and R-IHC for SLNs in patients with breast cancer. In cases 2 and 3, metastasis was not detected with HE staining, but micrometastasis was detected using R-IHC (Fig. 1). In particular, micrometastasis was not detected, even in the histopathological diagnosis based on HE staining. In case 4, metastasis was not detected with HE staining, but macrometastasis was detected using R-IHC. This result is consistent with the histopathological diagnosis. Among all the dissected lymph nodes in case 4, six macrometastases were found. Case 4 was invasive lobular carcinoma, and diffuse small cancer cells were detected. In case 5, ITCs were detected only through histopathological cytokeratin staining. This appears to be because different slices were analyzed; the intraoperative R-IHC slice had no metastases. After intraoperative pathological diagnosis, the frozen samples were fixed in buffered formalin and embedded in paraffin. The embedded samples were then cut into slices, which extended to the region containing the ITCs. However, this did not change the histological stage.

**Table 4:** Details of SLN diagnoses that differed between HE and CK staining.

case	Intraoperative		Permanent		type	Grade	ly	v	pT	pN	Number of metastases	p-Stage
	HE stain	R-IHC CK stain	HE stain	CK stain								
1	negative	micrometa	negative	micrometa	IDC	1	1	0	1a	1 mi	1	IB
2	negative	micrometa	micrometa	micrometa	IDC	2	1	1	1c	1 mi	1	IB
3	negative	micrometa	micrometa	micrometa	IDC	1	1	0	2	1 mi	1	IIB
4	negative	macrometa	macrometa	macrometa	ILC	1	1	0	2	2	6	IIIA
5	negative	negative	negative	ITC	ILC	2	1	0	1c	0 (i+)	1	IA

## Discussion

Conventional HE staining-based histopathological studies are limited, depending on the metastatic volume within a lymph node^11^. By contrast, the sensitivity of IHC analysis using an anti-cytokeratin antibody is sufficient to detect any metastasis, but the standard protocol requires 2–4 hours to complete, which makes clinical application for intraoperative diagnosis impractical. In the present study, our intraoperative R-IHC method, which takes only about 20 min to provide immunohistochemical information for intraoperative pathological diagnosis, was used to evaluate SNL metastasis in patients with breast cancer. Our findings show that R-IHC has better sensitivity and specificity than conventional HE staining alone. All nodes deemed positive using the permanent (standard) IHC procedure were also positive using the R-IHC procedure.

In this study, we detected more micrometastases than macrometastases using R-IHC, which is in contrast to conventional HE staining. However, many trials have concluded that the presence of micrometastases has no impact on patient outcome and no impact on whether or not to use systemic treatment or lymph node radiotherapy^8^. The ACOSOG Z 0011^12^ and AMAROS trials^13^ showed that axillary dissection could be safely omitted with the presence of 1–2 axillary macrometastases. So, what is the merit of intraoperative R-IHC method? One important advantage is cost reduction. R-ICH enables diagnosis as accurate as with OSNA, but at a cost that is less than a quarter of OSNA. The cost of IHC analysis is high because the primary antibodies are expensive. Therefore, reducing the amount of primary antibody needed for IHC can have a significant impact on cost. Toda *et al*. reported the concentration of primary antibody could be reduced by more than 90% by using R-IHC^9^. In this series, we used the same amount of primary and secondary antibodies as standard IHC, but in the future, we will likely reduce them. At that time, R-IHC will be particularly attractive in terms of cost.

The key feature of R-IHC is quick (within 20 min intraoperatively) and accurate detection of SLN metastases, but another benefit is its ability to shorten the time needed for microscopic diagnosis itself. When pathologists observe HE sections under a microscope, it is sometimes difficult to find metastatic lesions. By contrast, when using R-IHC, pathologists are able to readily find metastatic lesions, even within low-power fields (e.g. 40x). As a result, there is no uncertainty about whether there are undetected micrometastases or small cancer cells present. This reduces not only the time needed for intraoperative diagnosis, but also the effort necessary to find unclear lesions like micrometastasis, small cancer cells^14^, and artifacts of frozen section.

Using HE staining alone, it is difficult to detect small numbers of cancer cells within SLNs, such as occurs with invasive lobular carcinoma^15, 16^. In our series, SLN metastasis was missed by HE staining in one lobular carcinoma patient (case 4), but was detected as macrometastasis using R-IHC. In total, six macrometastases were detected because ALND was carried out intraoperatively following adjuvant chemotherapy. In cases of invasive lobular carcinoma, R-IHC was very useful for detecting small numbers of tumor cells. Nonetheless, in one patient (case 5), ITCs were detected only with histopathological cytokeratin staining (Table 4). This appears to be because a different slice was used; the intraoperative R-IHC slice had no metastases, but other slices used for permanent IHC contained ITCs. This did not change the histological stage, and it represents only 0.6% of the lymph node samples. Strictly speaking, it would be impossible to prove the permanent specimen did not contain evidence of metastasis, even if we had not detected it. However, because the resected lymph nodes were cut at 2-mm intervals, if there were any micrometastasis present, it would be possible to detect them. All lymph nodes were processed in the same way, but using histopathological IHC, ITCs were detected in only one lymph node (0.6%).

An important feature of IHC is that it enables confirmation of the pathological morphology. For example, IHC can reveal whether an SLN shows extranodal invasion, which is indicative of a worse prognosis or can be a predictor of non-SLN tumor burden^17–19^. In addition, Corben *et al*. reported a case in which IHC revealed the presence of endosalpingiosis in axillary lymph nodes^20^. This was a rare case but represents a potential pitfall that can result in report of a false positive, especially when using OSNA. Thus the morphology makes a crucial contribution to correct diagnosis of SLN metastasis.

There are several situations in which R-IHC could be potentially useful. First, it could be used for SLN biopsy after neoadjuvant chemotherapy. Neoadjuvant chemotherapy is the established treatment for locally advanced disease and is being used increasingly for early-stage breast cancer^21^. This therapeutic approach provides *in vivo* chemosensitivity testing and prognostic information. NSABP B-27 showed the utility of SLN biopsy in patients who received prior neoadjuvant chemotherapy^22^. Some trials also showed the utility of SLN biopsy in initially lymph node-negative (cN0) patients who received prior neoadjuvant chemotherapy^23, 24^. Moreover, other trials suggest that with appropriate patient selection, SLN biopsy could be used with initially lymph node-positive (cN+) patients who have received prior neoadjuvant chemotherapy^25, 26^. They also suggest eventually using a combined tracer to improve the false-negative rate, but that remains controversial. In addition, the best surgical approach in the axilla for the 20–40% of patients with initially positive lymph nodes (cN+) who, after neoadjuvant chemotherapy, are downstaged to a clinically negative lymph node status (ycN0) is unclear (SLN biopsy or ALND or radiotherapy)^27, 28^. NSABP B-18 showed that detection of micrometastases after neoadjuvant chemotherapy is a poor prognostic factor^27^, and there is limited evidence of an established surgical approach, especially in patients with initially positive lymph nodes (cN+) who are downstaged to a clinically negative lymph node status (ycN0) after neoadjuvant chemotherapy. In such situations, R-IHC could potentially be useful for accurate intraoperative axillary lymph node diagnosis.

Second, for patients with hormone receptor-positive breast cancer who need multigene assays to confirm chemotherapy sensitivity. A diagnostic system comprising a 95-gene classifier was developed for predicting the prognosis of node-negative and ER-positive breast cancer patients using already published DNA microarray data, which classified the patients into low-risk and high-risk groups. The system enables one to determine who is likely to benefit from postoperative adjuvant chemotherapy. Because this approach requires tumor tissue obtained at surgery to be snap frozen in liquid nitrogen and kept at −80 °C until use, diagnosis of SLN metastasis must be made intraoperatively, and R-IHC could be applicable^29–31^.

Third, with R-IHC the specimen is preserved, which is helpful when there is a need for further examination – for example, determination of the subtype of a lymphatic metastatic lesion or any other additional histopathological or molecular investigation. Even if a patient has multiple breast carcinomas and they are different types, we should be able to correctly diagnose the subtype or histological type of SLN metastases using R-IHC. And sometimes the R-IHC results could help to determine the subsequent systemic therapy in advanced or metastatic breast cancer patients, since there is sometimes inconsistency between the breast carcinoma and lymph node metastases^32^.

In this series, we applied R-IHC to intraoperative SLN biopsy in patients with breast cancer. The limitation of this study, the number of cases will be small in the breast cancer research area, however it seems to be possible to show the relevant and usefulness for clinical application of this cutting-edge technology. This study was conducted at a single institute. Further investigation in a multi-institutional collaborative prospective study will be needed to confirm the utility of this method. But for now, R-IHC appears to be a cost-effective and clinically applicable method for diagnosis of breast cancer and SLN metastasis.

## Patients and Methods

### Patients

**Figure 2:**
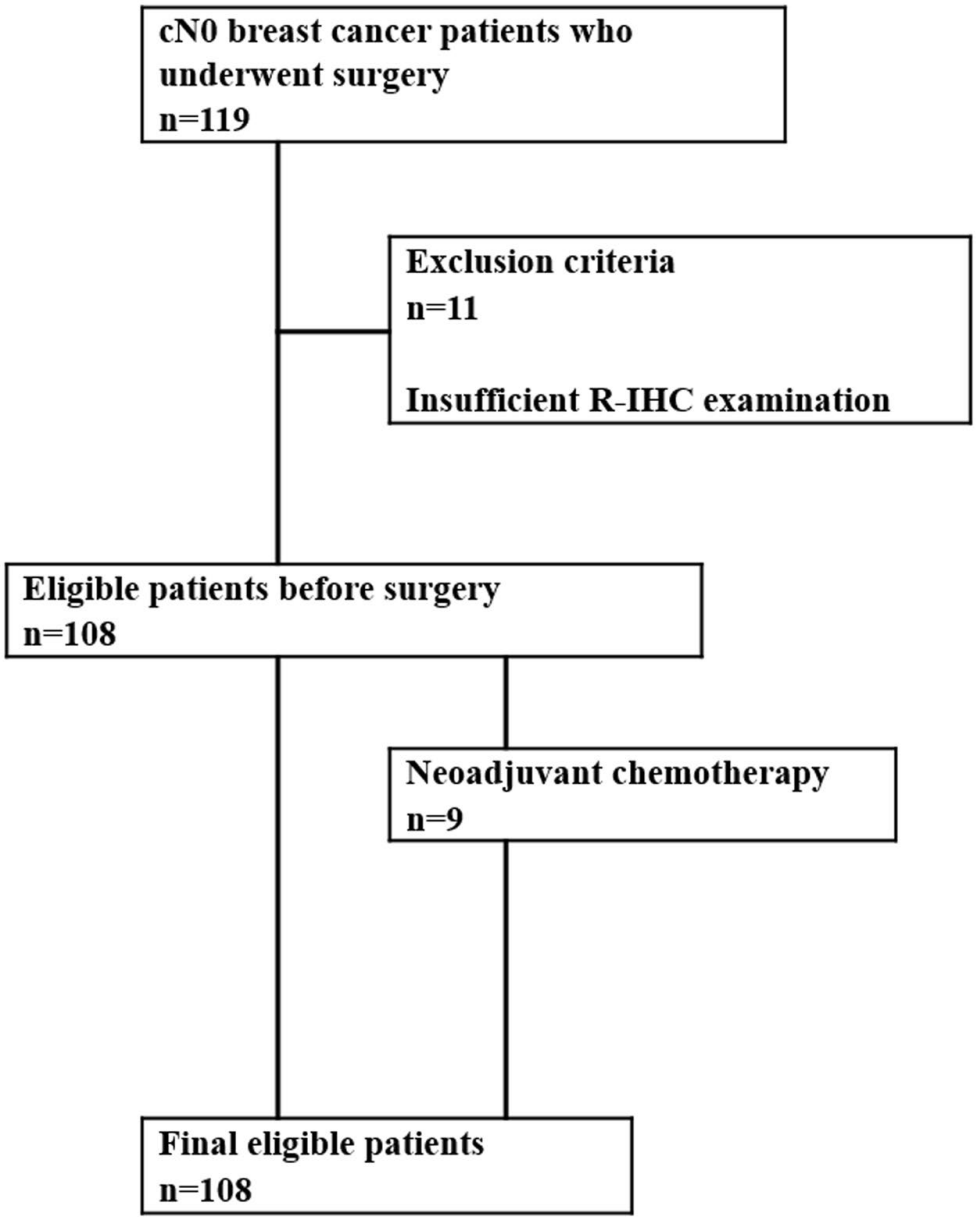
Diagram for patients selection.

One hundred-eight patients with breast cancer were enrolled in this study between July 2014 and January 2017. This prospective cohort study was approved by the Institutional Review Board at Akita University School of Medicine and University Hospital (Permit number: 896). Informed consent was obtained from all patients after discussion of the general risks and benefits of surgery for breast cancer. The methods in this study were carried out in accordance with the approved guidelines. Patients were selected as shown in the diagram in Fig. 2. A total of 119 clinically node-negative (cN0) patients with breast cancer underwent surgery in our institute. From among those, 108 eligible patients were enrolled in the study. Eleven patients were excluded from this study because only one pathologist, not two, evaluated the R-IHC staining of the dissected SLNs. No eligible patients declined participation in this study.

After preoperative evaluation, the patients were taken to an operating room, and the standard preparations were made for breast surgery. Patients were administered lymphoscintigraphies before the operation. Breast conserving surgery or mastectomy with SLN biopsy was then performed using the combined blue dye and radioisotope ((99 m) Tc-phytate) method. Axillary lymph node dissection (ALND) was carried out for intraoperative detection of SLNs showing macrometastasis. The 160 intraoperatively dissected SLNs were sectioned, stained with HE and immunohistochemically labeled with anti-cytokeratin antibody using R-IHC procedures. The sections were then independently examined and evaluated by two pathologists. After the operation, HE and standard IHC staining for permanent tissues were performed. Adjuvant chemotherapy was considered based on the subtype and histopathological diagnosis in each case.

### Tissue preparation

For intraoperative rapid pathological diagnosis, resected axillary lymph nodes were cut at 2-mm intervals, then immediately embedded in optimum cutting temperature (O.C.T.) compound (Tissue-Tek^®^, Sakura Finetek Japan Co., Ltd., Tokyo, Japan), frozen for 30 s in liquid acetone at −80 °C using a frozen specimen block preparation system (Histo-Tek Pino^®^, Sakura Finetek Japan Co., Ltd., Tokyo, Japan), and transferred to a cryostat for sectioning (Leica CM 1900^®^, Wetzlar, Germany).

### R-IHC device and procedure

**Figure 3:**
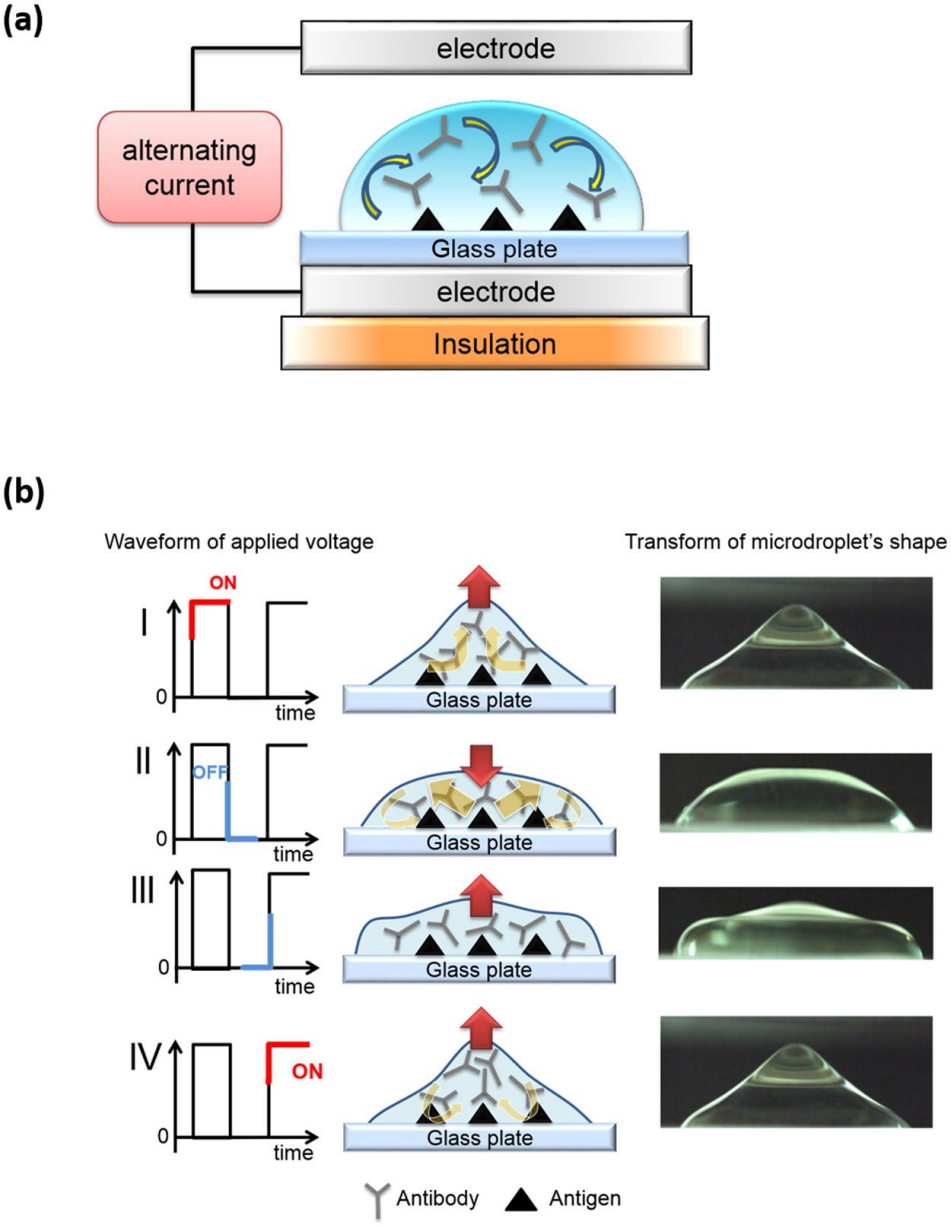
Schema of the device used to apply a high-voltage, low-frequency AC electric field. The slide was placed between the electrodes, and a high-voltage (4 KV), low-frequency (5 Hz) AC current was applied (**a**). The schema shows the changes within a microdroplet as the voltage is switched on and off in a time series (I → II → III → IV), which mixes the antibodies (**b**).

We have developed a device that reduces the time required for IHC analysis. Its mechanism was previously described in detail^9, 33^. With this R-IHC system (Histotech-RIHC^®^, Sakura Finetek Japan Co., Ltd.), we apply a high-voltage, low-frequency AC electric field to lymph node sections while they are incubating with the antibodies (Fig. 3a). The antibodies are mixed within microdroplets as the voltage is switched on and off in a time series. The resultant coulomb force stirs the antibody solution on the sections, and the opportunity for contact between the antibody and antigen is increased because as the voltage is turned on and off, the microdroplets’ shape is transformed (Fig. 3b). This greatly reduces the time required for the antigen-antibody reaction. For each specimen, 4-μm-thick frozen sections were cut at 2-mm intervals, then placed on slides and subjected to the staining procedure. Details of the staining method are listed in Table 2. Briefly, the slides were incubated first for 5 min with anti-cytokeratin (MNF116, 1:200 dilution, Abcam plc, Cambridge, UK) on the R-IHC device system. The primary antibody was then detected by incubating the slides for 5 min with a one-step simple stain system (Simple stain MAX-PO (MULTI)^®^, Nichirei Corporation, Tokyo, Japan), after which the slides were developed using 3,3′ diaminobenzidine (DAB, Dako, Tokyo, Japan) and counterstained with hematoxylin. Finally, the slides were dehydrated and mounted on coverslips. Using this method we can complete IHC analyses within about 20 min.

### HE and standard IHC for permanent tissues

After intraoperative pathological diagnosis, the specimens were fixed in 10% buffered formalin and embedded in paraffin. Three-micrometer-thick sections of formalin-fixed paraffin-embedded samples were incubated with Paraffin Stretcher (Sakura Finetek Japan, Tokyo, Japan) at 50 °C overnight, then stained using HE and IHC. IHC staining for cytokeratin (MNF116, 1:200 dilution, Abcam plc, Cambridge, UK) was performed using the labeled streptavidin biotin method with a Ventana BenchMark XT immunostainer (Ventana Medical Systems, Tucson, AZ, USA).

### Histopathological evaluation

Samples from all dissected lymph nodes were sectioned, conventionally stained with HE and immunohistochemically labeled with anti-cytokeratin using R-IHC and standard IHC and examined by two pathologists. Macrometastasis was defined as a metastatic lesion larger than 2 mm in diameter, while micrometastasis was defined as a metastatic focus greater than 0.2 mm in diameter, but with an upper limit of 2 mm. Isolated tumor cells (ITCs) (single cells or cell deposits) were defined as a focus of metastatic carcinoma measuring no larger than 0.2 mm or with fewer than 200 cells. A result was considered positive for lymph node metastasis if either macrometastasis or micrometastasis was detected on the slides. ITCs were classified as negative lymph node metastasis in this study, based on the 7th edition of American Joint Committee on Cancer Staging Manual^34^.
